# CCL2-driven inflammation increases mammary gland stromal density and cancer susceptibility in a transgenic mouse model

**DOI:** 10.1186/s13058-016-0796-z

**Published:** 2017-01-11

**Authors:** Xuan Sun, Danielle J. Glynn, Leigh J. Hodson, Cecilia Huo, Kara Britt, Erik W. Thompson, Lucy Woolford, Andreas Evdokiou, Jeffrey W. Pollard, Sarah A. Robertson, Wendy V. Ingman

**Affiliations:** 1 Discipline of Obstetrics and Gynaecology, School of Medicine, University of Adelaide, Adelaide, Australia; 2 The Robinson Research Institute, University of Adelaide, Adelaide, Australia; 3 Discipline of Surgery, School of Medicine, The Queen Elizabeth Hospital, University of Adelaide, DX465702, 28 Woodville Rd, Woodville, 5011 Australia; 4 The University of Melbourne Department of Surgery, St Vincent’s Hospital Melbourne, Fitzroy, Australia; 5 Metastasis Research Laboratory, Peter MacCallum Cancer Centre, Melbourne, Australia; 6 The Sir Peter MacCallum Department of Oncology, University of Melbourne, Melbourne, Australia; 7 Institute of Health and Biomedical Innovation, School of Biomedical Sciences, Queensland University of Technology and Translational Research Institute, Queensland, Australia; 8 School of Veterinary Sciences, University of Adelaide, Roseworthy, SA Australia; 9 MRC and University of Edinburgh Centre for Reproductive Health, Queen’s Medical Research Institute, University of Edinburgh, Edinburgh, UK

**Keywords:** Mammary gland, Development, Macrophage, Chemokine (C-C motif) ligand 2, Mouse model, Mammographic density

## Abstract

**Background:**

Macrophages play diverse roles in mammary gland development and breast cancer. CC-chemokine ligand 2 (CCL2) is an inflammatory cytokine that recruits macrophages to sites of injury. Although CCL2 has been detected in human and mouse mammary epithelium, its role in regulating mammary gland development and cancer risk has not been explored.

**Methods:**

Transgenic mice were generated wherein CCL2 is driven by the mammary epithelial cell-specific mouse mammary tumour virus 206 (MMTV) promoter. Estrous cycles were tracked in adult transgenic and non-transgenic FVB mice, and mammary glands collected at the four different stages of the cycle. Dissected mammary glands were assessed for cyclical morphological changes, proliferation and apoptosis of epithelium, macrophage abundance and collagen deposition, and mRNA encoding matrix remodelling enzymes. Another cohort of control and transgenic mice received carcinogen 7,12-Dimethylbenz(a)anthracene (DMBA) and tumour development was monitored weekly. CCL2 protein was also quantified in paired samples of human breast tissue with high and low mammographic density.

**Results:**

Overexpression of CCL2 in the mammary epithelium resulted in an increased number of macrophages, increased density of stroma and collagen and elevated mRNA encoding matrix remodelling enzymes lysyl oxidase (LOX) and tissue inhibitor of matrix metalloproteinases (TIMP)3 compared to non-transgenic controls. Transgenic mice also exhibited increased susceptibility to development of DMBA-induced mammary tumours. In a paired sample cohort of human breast tissue, abundance of epithelial-cell-associated CCL2 was higher in breast tissue of high mammographic density compared to tissue of low mammographic density.

**Conclusions:**

Constitutive expression of CCL2 by the mouse mammary epithelium induces a state of low level chronic inflammation that increases stromal density and elevates cancer risk. We propose that CCL2-driven inflammation contributes to the increased risk of breast cancer observed in women with high mammographic density.

## Background

Development and function of the mammary gland is dependent upon dynamic interactions between the mammary gland epithelium and surrounding stroma. Macrophages are a major component of the stroma, and have the capacity to direct a diverse range of biological processes necessary for mammary gland development, including cell proliferation, differentiation, phagocytosis, and tissue remodelling [1–6]. In addition to roles in healthy development, macrophages may also affect cancer susceptibility [7, 8]. Macrophages suppress cancer development as part of innate and adaptive anti-tumour immune responses, through recognition of DNA damaged cells, phagocytosis and antigen presentation to tumour-specific T cells [9–11]. On the other hand, macrophages can promote the proliferation and survival of cancer cells, facilitate tumour cell invasion, increase angiogenesis, and upregulate the production of pro-tumourigenic factors, and thus promote tumour progression [12–14]. In order to exert this wide range of effects in healthy development and cancer susceptibility in the mammary gland, macrophages respond to a variety of different signals in their local microenvironment, including cytokines emanating from other cell types, and components of the extracellular matrix.

CC-chemokine ligand 2 (CCL2), also known as monocyte chemotactic protein 1, is a small pro-inflammatory cytokine and is a highly potent chemoattractant for monocytes and macrophages to sites of tissue injury and inflammation [15, 16]. CCL2 can be highly expressed by both the tumour and surrounding stromal cells in breast carcinomas [17–19]. CCL2 expression in breast carcinomas is highly associated with macrophage infiltration, and its expression is correlated with poor prognosis in breast cancer patients [18–21]. Studies in mice have implicated CCL2 of epithelial tumour cell origin, and macrophages expressing the CCL2 receptor, CCR2, as critical factors in metastasis of breast cancer to the bone and lungs [19, 22]. These studies suggest that epithelial cell-derived CCL2 in carcinomas might promote tumour invasion and metastasis through increased infiltration of macrophages into tumours.

Less understood is the role of CCL2 in healthy breast development and how this may relate to the risk of cancer initiation. CCL2 protein is detected in human breast epithelium [17, 18] and may be responsible for macrophage recruitment under specific conditions. To investigate the effect of CCL2 in mammary gland development and function, we developed a transgenic mouse model *Mmtv-Ccl2*, whereby CCL2 is constitutively expressed by the mammary epithelium. We found that these mice exhibit increased macrophage recruitment to the mammary gland, perturbed mammary morphogenesis at the proestrus phase of the ovarian cycle and increased abundance of stroma. Overexpression of CCL2 also increased susceptibility to 7,12-Dimethylbenz(a)anthracene (DMBA)-induced mammary tumour development. These key features of the mouse model resemble breast tissue with high mammographic density in women, which is associated with increased stroma and collagen deposition [23, 24], increased immune cell abundance [25], and a fourfold to six-fold increased risk of breast cancer when adjusted for body mass index (BMI) and age [26]. A paired sample analysis of human breast tissue of high and low mammographic density showed that CCL2 protein is higher in tissue with high mammographic density. Combined, these findings suggest that high mammographic density and the associated increased cancer risk may be the result of CCL2-driven inflammation.

## Methods

### Mice

Animal experiments were approved by the University of Adelaide Animal Ethics Committee and were conducted in accordance with the Australian Code of Practice for the Care and Use of Animals for Scientific Purposes (7th ed., 2004). All mice were maintained in specific pathogen-free conditions with controlled light (12-h light, 12-h dark cycle) and temperature at the Laboratory Animal Services Medical School facility. Food and water were provided ad libitum. In the experiments we utilised *Mmtv-Ccl2* transgenic mice on an FVB background and non-transgenic FVB mice as controls. Estrous cycle stage was determined by analysis of vaginal smears as described previously [27]. Estrous cycles were tracked for at least 28 days.

### Generation of Mmtv-Ccl2 transgenic mice

The *Mmtv-Ccl2* transgenic mouse, in which the mouse mammary tumour virus 206 promoter (*Mmtv*) long terminal repeat (generously provided by Dr. William Muller, McGill University) constitutively drives the expression of the chemokine (C-C motif) ligand 2 (*Ccl2*) mRNA, was generated at the Albert Einstein College of Medicine (New York, USA) by Jiufeng Li. The *Mmtv-Ccl2* expression cassette was constructed by insertion of a 3.2-kb EcoRI fragment of the mouse genomic *Ccl2* cDNA from the construct pMMJE + 20 [28] into the EcoRI site of the plasmid MMTV-SV40-Bssk, which contain regulatory elements of the *Mmtv* promoter followed by the SV40 poly A site and the ampicillin-resistance gene (*ampr*) [29]. DH5-α cells were transformed with this construct by heat shock and selected for ampicillin resistance. DNA was extracted from the successfully transformed cells and the *Mmtv-Ccl2* expression cassette was released as a PVUII fragment of 8 kb in size. This purified *Mmtv-Ccl2* expression cassette was micro-injected into zygotes of FVB mice, which were then transferred into pseudopregnant recipient mothers.

**Fig. 1:**
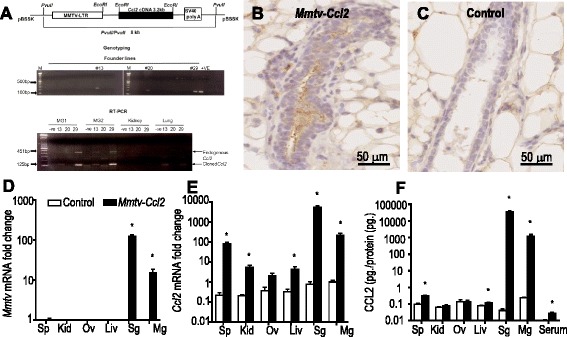
Generation of mouse mammary tumour virus 206 transgenic mice (*Mmtv-Ccl2*). The *Mmtv-Ccl2* expression cassette was detected in three of twenty-nine mice by PCR screening and the expression of both endogenous genomic and cloned *Ccl2* mRNA in different tissues was measured by RT-PCR with product sizes of 451 bp and 125 bp, respectively (**a**). CCL2 protein detected by immunohistochemistry in the mammary gland from female offspring from founder mouse #29 (**b**) compared to non-transgenic control (**c**). RNA encoding MMTV (**d**) and CCL2 (**e**), and CCL2 protein (**f**) were quantified in spleen (*Sp*), kidney (*Kid*), ovary (*Ov*), liver (*Liv*), salivary gland (*SG*) and mammary gland (*MG*) from *Mmtv-Ccl2* and non-transgenic control mice. Abundance of mRNA was normalised to *Actb* expression, and is given in arbitrary units where the average of the non-transgenic mammary gland control is 1; *n* = 5 per group. Data are presented as mean + SEM with statistical analysis conducted using the unpaired t test, *p < 0.05

Twenty-nine offspring were generated, and three founder lines (*Mmtv-Ccl2* #13, #20 and #29) were identified by PCR screening using a primer pair (forward: 5’-CGT CCA GAA AAC CAC AGT CA -3’; reverse: 5’-CCG CTC GTC ACT TAT CCT TC-3’) covering the *Mmtv* promoter sequence, which produced a product size of 196 bp (Fig. 1a). All three founders were cross-bred with background strain FVB mice and the genotypes of all progeny were confirmed by PCR. One female pup was selected from each founder line and the expression of *Ccl2* mRNA from different tissues was measured by RT-PCR using a primer pair that spanned both endogenous genomic and exogenous cloned *Ccl2* (forward: 5-CCC AAT GAG TAG GCT GGA GA-3’; reverse: 5’-TCT GGA CCC ATT CCT TCT TG-3’) and produced product sizes of 451 bp and 125 bp, respectively (Fig. 1a). The offspring from founder 29 exhibited highest expression of mRNA encoding *Ccl2* in the mammary gland and was chosen for further analysis. A homozygous *Mmtv-Ccl2* transgenic mouse line was successfully established from founder 29 and maintained for over five generations on a FVB background.

### DMBA-induced tumour susceptibility

Susceptibility of mice to mammary gland tumours was investigated using the DMBA-induced mammary tumour model. This model is superior for this purpose over other mouse mammary tumour models, such as transgenic expression of polyoma middle T oncogene targeted to the mammary mouse mammary gland, because tumour development does not occur with 100% penetrance, enabling quantification and statistical analysis of cancer susceptibility. *Mmtv-Ccl2* and FVB mice received DMBA in sesame oil (1 mg/ml) weekly by oral gavage for 6 weeks from 6 weeks of age. General health and signs of tumour development were monitored weekly. To detect tumours, the mammary glands were gently palpated. Mice were killed when tumours were detected and the tumours dissected, formalin-fixed and paraffin-embedded.

### Tissue morphology and histology

To analyse the morphology of the mammary gland, mammary glands were dissected from mice and spread on a glass slide. The mammary gland whole mounts were incubated in Carnoy’s fixative and stained in carmine alum as described previously [30]. To analyse mammary gland histology, mammary glands were dissected from mice and fixed in 4% paraformaldehyde (PFA) (Sigma-Aldrich) for 24 h at 4 °C. Paraffin-embedded tissue was cut on a Leica Rotary Microtome (Leica Microsystems) and placed onto SuperFrost Plus slides. Slides were dried overnight at 37 °C. Blocks and sections were stored at room temperature prior to staining.

Haemotoxylin and eosin staining was performed on paraffin-embedded sections. The sections were dewaxed in Safsolv (Ajax, Finechem, Australia) and passed sequentially through 100%, 95%, 80% and 70% ethanol for rehydration. The sections were stained with haemotoxylin and counterstained with eosin and then mounted on coverslips.

Masson’s Trichrome stain was used for detection of collagen fibres in paraffin-embedded mouse mammary gland tissue (all reagents were from Sigma-Aldrich). Slides were placed in Bouin’s fluid overnight at room temperature. The following day, slides were placed in Weigert’s Haematoxylin working solution (50% A and 50% B) for 10 minutes at room temperature and immersed in 80% ethanol before washing with MQ water. Slides were placed in Biebrich Scarlet solution for 2 minutes and immersed in 5% phosphotungstic acid for 15 minutes followed by immersing in Light green solution for 10 minutes. Slides were immersed in 1% glacial acetic acid for 5 minutes prior to dehydration, clearing and mounting.

### Immunohistochemistry

Proliferating cells were identified by bromodeoxyuridine (BrdU) immunostaining of paraffin-embedded mammary gland tissue. One hour prior to sacrifice, mice received an intraperitoneal (i.p.) injection of 100 μl of 10 mg/ml BrdU (Sigma). Sections were stained for BrdU incorporation into DNA using a BrdU In-Situ Detection Kit (BD Biosciences Pharmingen) according to the manufacturer’s instructions.

Apoptotic cells were identified by terminal deoxynucleotidyl transferase dUTP nick end labelling (TUNEL) staining of paraffin-embedded mammary gland tissue. Sections were stained with the In-Situ Cell Death Detection Kit (Roche) according to the manufacturer’s instructions. Only BrdU and TUNEL positive cells clearly located within the ductal or alveolar epithelium were included in the analysis.

Macrophage abundance was determined by F4/80 antibody staining. Five micrometer paraffin-embedded sections mounted on glass slides were incubated with rat anti-F4/80 monoclonal antibody (1:100 dilution; overnight at 4 °C) (Caltag Laboratories, Burlingame, CA, USA) followed by biotinylated rabbit anti-rat IgG (1:200 dilution; 40 minutes at room temperature) (Vector Laboratories) and ABC Elite kit (Vector Laboratories) with 3,3 diaminobenzadine (DAB) peroxidase (DAKO, Denmark).

CCL2 was detected with polyclonal rabbit anti-mouse CCL2 (Santa Cruz Biotechnology, CA, USA) in 5-μm paraffin-embedded sections mounted on glass slides. For antigen retrieval, slides were placed in 10 mM sodium citrate buffer (pH 6) and brought to 90 °C in a water bath for 20 minutes and washed two times in PBS for 3 minutes each. Following CCL2 antibody incubation (1:50 dilution; overnight at 4 °C), sections were incubated with goat anti-rabbit HRP (1:200 dilution; Dako) for 60 minutes at room temperature. The detection of bound antibody was performed using DAB according to the manufacturer’s instructions.

Collagen-1 was detected using Alexa Fluor® 594 goat-anti rabbit IgG (Chemicon, MA, USA, 1:800 dilution). Slides stained with secondary antibodies only or with isotype-matched antibody were included as negative controls. All sections were mounted in fluorescent mounting medium (Dako, Glostrup, Denmark) with 4’,6-diamidino-2-phenylindol (DAPI) (Sigma, St Louis, USA) and were stored at 4 °C in the dark until image capture.

### Histology, pathology and immunohistochemistry quantification

Fluorescence images of collagen-1 and TUNEL-positive apoptotic epithelial cells were captured and collected using FV10i Confocal Microscope (Olympus, USA) with laser-power and photomultiplier settings kept constant for all experiments, and images were captured at × 60 magnification. Non-fluorescent stained tissue sections were captured as a digital image using a Nanozoomer 1.0 (Hamamatsu, Shizouka, Japan) at a zoom equivalent to a × 40 objective lens. Whole mount images of mammary glands were captured by MZ16 FA-Stereo microscope (Leica, The University of Adelaide, SA, Australia).

An assessor blinded to mouse genotype performed all quantification analysis. To determine the extent of ductal branching morphogenesis in whole-mounted mammary glands, the three longest ducts from each mammary gland were selected, the number of branch points on each duct was counted manually, and a mean value of three ducts per mammary gland were calculated and expressed as branch points/mm.

To quantify the extent of alveolar development in H&E-stained sections, epithelium was categorized as ductal (single epithelium layer) or alveolar (clusters of epithelial structures containing alveolar lumens) and the numbers of ductal and alveolar epithelial structures were counted manually as described previously [31, 32]. The total number of alveolar buds was expressed as percentage of total epithelial structures (ductal plus alveolar).

To determine the ratio of stroma and epithelium area within the mammary gland in H&E-stained sections, five ductal epithelium and alveolar epithelium regions were randomly chosen for quantification. The area of stroma and epithelium was measured and represented as stroma area (mm^2^)/epithelium area (mm^2^). To determine the number of epithelial cells within the ductal epithelium, the number of haematoxylin-positive nuclei was counted.

To determine the deposition of collagen around mammary epithelium, the amount of fibre stain (green) around five randomly chosen ductal epithelia and alveolar epithelia was quantified. To determine the number of proliferating epithelial cells and apoptotic epithelial cells, the number of BrdU-positive cells and TUNEL-positive cells within the epithelium was counted respectively. The number of F4/80-positive cells in the mammary gland was counted and only positive cells with visible haematoxylin-stained nuclei were included. For F4/80 staining, F4/80-positive macrophages were distinguished from F4/80-stained eosinophils on the basis of nuclear morphology. All results were expressed as positive cells/mm^2^ or percentage of positive cells. The mean density of positive cells within the five ductal and the five alveolar stroma regions was calculated, and grouped by mouse genotype.

The thickness of total collagen (indicated by red arrows) around each selected ductal epithelium was measured, quantified and represented as collagen thickness/μm (E). The intensity of collagen I was also measured around each selected ductal epithelium, and was quantified and represented as collagen I density (arbitrary unit) (F). The mean fluorescence intensity (mean grey-scale value) of collagen I was determined and analysed using ImageJ software and a mean value of five randomly chosen epithelial ducts was calculated.

Haemotoxylin and eosin stained sections of DMBA-induced mammary tumours were assessed by a veterinary pathologist (LW). The tumours were classified and scored for mitotic index and inflammation grade. Mitotic index was determined by counting the number of mitotic cells in a 10× field of view. Inflammation was scored on a scale of 0–5, which took into account degree of inflammation and degree of dispersion as either 0 (none), 1 (mild, focal), 2 (mild, multifocal), 3 (moderate, focal), 4 (moderate, multifocal) or 5 (marked, diffuse or multifocal).

### CCL2 ELISA

All tissues and serum were collected under sterile conditions, and the tissue was snap frozen in liquid nitrogen and stored at −80 °C until processing. Protein was extracted from tissues in RIPA buffer containing Complete Protease Inhibitor Cocktail Tablets (Roche, Basel, Switzerland). Protein concentration of each sample was quantified using the BCA protein assay (Thermo Scientific) following the manufacturer’s instructions. The limit of detection was between 20 μg/ml and 2000 μg/ml.

A CCL2-specific sandwich ELISA (eBioscience, San Diego, CA, USA) was used to quantify mouse CCL2 protein in different tissues, according to the manufacturer’s instructions. Briefly, an anti-mouse CCL2 antibody was coated onto a 96-well microtitre plate to capture CCL2 from either recombinant standard or assay samples. A biotinylated anti-mouse CCL2 detection antibody was then used and bound antibody was quantified by the addition of streptavidin-conjugated horseradish peroxidase (HRP), followed by the addition of a chromagen substrate. After incubation for 20 minutes at room temperature, the substrate product was acidified by the addition of 50 μl of 1 M HCl, and absorbance at 450 nm (reference wavelength 570 nm) was measured using a Benckmark™ microplate reader (Bio-Rad Laboratories). The concentration of CCL2 within tissues and serum was then calculated from a standard curve (five-parameter logistic curve) using known concentrations of recombinant CCL2. This assay was reported by the manufacturer to have a minimum detection limit of 15 pg/ml, with intra-assay and inter-assay precision of approximately 5%. The concentration of CCL2 in each sample is expressed relative to the protein concentration of the tissue, e.g. CCL2 (pg)/protein (pg).

### mRNA expression

Spleen, mammary gland, kidney, ovary and salivary gland were dissected from *Mmtv-Ccl2* and FVB mice to quantify expression of *Ccl2*, *Mmtv*, *Mmp2*, *Mmp9*, *Lox*, *Timp1*, *Timp2* and *Timp3* mRNA expression. Total RNA was extracted using Trizol (Invitrogen) and treated with DNase (DNA free; Ambion), then first-strand cDNA was reverse transcribed from 3 μg random hexamer-primed RNA employing a Superscript-III Reverse Transcriptase kit. Primer pairs specific for published cDNA sequences were designed using Primer Express version 2 Software (Applied Biosystems, Foster City, CA, USA). The following primer pairs were used to detect specific cDNAs: *Actb* 5' GTG TGA CGT TGA CAT CCG TAA AG 3' CTC AGG AGG AGC AAT GAT CTT GAT; *Ccl2* 5' CCA GCA AGA TGA TCC CAA TGA 3' TCT CTT GAG CTT GGT GAC AAA AAC; *Mmtv* 5' CGT CCA GAA AAC CAC AGT CA 3' CCG CTC GTC ACT TAT CCT TC; *Mmp2* 5' CAA GTT CCC CGG CGA TGT C 3' TTC TGG TCA AGG TCA CCT GTC; *Mmp9* 5' CAG ACG TGG GTC GAT TCC A 3' TGT CTC GCG GCA AGT CTT C; *Timp1* 5' AGT CCC AGA ACC GCA GTG AA 3' AGT ACG CCA GGG AAC CAA GA; *Timp2* 5' GAG CCT GAA CCA CAG GTA CCA 3' GTC CAT CCA GAG GCA CTC ATC; *Timp3* 5’ CTT CTG CAA CTC CGA CAT CGT 3’ TCA GAG GCT TCC GTG TGA ATG; *Lox* 5’ TGC CAG TGG ATT GAT ATT ACA GAT GT 3’ AGC GAA TGT CAC AGC GTA CAA. PCR amplification was performed in duplicate in an ABI Prism 7000 Sequence Detection System (Applied Biosystems) using SYBR Green PCR Master Mix (Applied Biosystems). Reaction products were analysed by dissociation curve profile and by 2% agarose gel (wt/vol) electrophoresis. Assay optimization and validation experiments were performed to define the amplification efficiency of each primer pair as described previously [33]. Messenger RNA abundance values were normalized independently to *Actb* mRNA expression, and data are plotted as relative expression in arbitrary units, adjusted such that the mean of the wild-type control group is assigned a value of 1.

### Quantification of CCL2 in paired human breast tissue samples

To investigate the relationship between mammographic density and CCL2, immunohistochemistry to detect CCL2 was conducted on paired breast tissue samples of high mammographic density (HMD) and low mammographic density (LMD). This approach has been described previously [23, 25], and offers excellent scope to study histological parameters associated with HMD in small sample sizes, as paired-sample statistical analysis can be applied.

Briefly, ethics approval from the Peter MacCallum Human Research Ethics Committee (number 08/21) and St. Vincent’s Hospital, Victoria was obtained and the study conducted in accordance with the Australian National Statement on Ethical Conduct in Human Research. Breast tissue was collected from women undergoing prophylactic mastectomy for breast cancer prevention consented to the study through the Victorian Cancer Biobank (VCB 10010). These women had a confirmed BRCA1/2 carrier status, a past history of breast cancer in the other breast or a family history of two or more first-degree or second-degree relatives with breast cancer who were diagnosed before the age of 50 years. Resected breast tissue was transferred immediately on ice to the pathology department upon completion of mastectomy, where pathologists resected slices of breast tissue using a sterile technique. X-rays of those slices were taken by a breast radiographer using uniform radiological parameters and were assessed against a calibration ruler for selection of HMD and LMD regions. The tissue slice was transferred to a biosafety level-2 hood, where areas that appeared white on X-rays were selected and removed using sterile blades and defined as HMD regions, and areas that appeared black were similarly selected, removed and classified as LMD regions. The HMD and LMD regions were subjected to routine formalin fixation and paraffin embedding.

Paraffin-embedded human breast tissue sections were placed on a hotplate at 60 °C for 60 minutes before dewaxing in two 5-minute washes in Safsolv and gradually passed through 100%, 95%, 80% and 70% ethanol for rehydration as described previously [23, 25]. Before sections were incubated with mouse anti-human CCL2 antibody (R&D systems) for 30 minutes at room temperature, sections were placed in Dako EnVision™ low pH antigen retrieval solution (Dako) and brought to 90 °C in a water bath for 20 minutes followed by Envision™ wash buffer and endogenous peroxidase block. After primary antibody incubation, sections were incubated with Dako EnVision™ HRP (ready-to-use, Dako) for 30 minutes at room temperature and washed in Envision™ wash buffer for 5 minutes. The detection of bound antibody was performed according to the manufacturer’s instructions. Tissue sections were counterstained with haematoxylin prior to dehydration, and cleared and mounted as described above. Slides stained with isotype-matched primary antibody were included as negative controls.

Stained tissue sections were captured as a digital image using a Nanozoomer 1.0 (Hamamatsu, Shizouka, Japan) at a zoom equivalent to a × 40 objective lens. All quantification analysis was performed blinded. Three epithelium clusters were randomly selected from each section for quantification. To determine the intensity of CCL2-positive staining within the epithelium of each cluster, staining was quantified using the IHC profiler within Image J analysis software. The percentage of positive staining was determined by combining percentage of high, medium and low positive staining within each selected epithelium measured by the IHC profiler and the mean value of three clusters per patient was calculated.

### Statistical analysis

Data were assessed for normal distribution with a Shapiro-Wilk normality test using GraphPad Prism 5 (GraphPad software Inc, San Diego, CA, USA) or SPSS Statistics Version 17.0 (IBM Corporation, Armonk, NY, USA). Normally distributed data were analysed using the unpaired *t* test with the exception of paired high and low mammographic density samples, which were analysed by paired *t* test. Not normally distributed data were analysed using the Mann–Whitney *U* test. Data are presented as the mean ± SEM (standard error of mean). Kaplan-Meier survival curves were generated using SPSS Statistics Version 17.0 to analyse survival function and the log rank test was used to compare different Kaplan–Meier curves between groups. Tumour incidence was analysed by the chi-squared test. The difference between control and transgenic groups was considered statistically significant if *p* < 0.05 and is indicated on the figures by an asterisk.

## Results

### Elevated expression of Ccl2 mRNA and protein in Mmtv-Ccl2 mice

To determine whether CCL2 abundance was elevated in transgenic mice, mRNA encoding the MMTV promoter and CCL2, and CCL2 protein, were investigated in offspring from mouse founder line #29. CCL2 protein was clearly detectable in the lumen of the mammary gland of *Mmtv-Ccl2* mice by immunohistochemistry, and was substantially elevated compared to the very low abundance in non-transgenic control mice (Fig. 1b and c). No positive staining was observed in the mammary epithelium stained with isotype-matched negative control antibody (not shown). Messenger RNA encoding MMTV was virtually undetectable in the spleens, kidneys, ovaries and livers of all mice, and in the mammary gland and salivary gland of non-transgenic control mice (Fig. 1d). However, abundance of mRNA encoding MMTV was detectable in the mammary gland and salivary gland of *Mmtv-Ccl2* mice and was 15-fold and 125-fold higher, respectively, compared to control mice.

Consistent with previous studies using CCL2 expression cassettes driven by different promoters [34, 35], elevated abundance of CCL2 was observed not only in the tissue of interest, but also in other tissues and in the blood of *Mmtv-Ccl2* mice. Abundance of *Ccl2* mRNA was significantly elevated in the spleen, kidney, salivary gland, mammary gland and liver of *Mmtv-Ccl2* mice compared to control mice (Fig. 1e). The abundance of CCL2 protein was also elevated in the spleen, liver, salivary gland, and mammary gland and in the serum of *Mmtv-Ccl2* mice compared to control mice (Fig. 1f).

### CCL2 overexpression increases recruitment of macrophages to the mammary gland

**Fig. 2:**
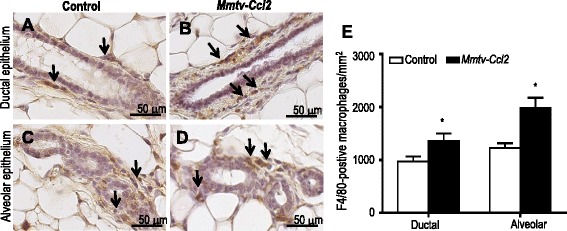
The effect of CCL2 overexpression on macrophage abundance and location within and around mammary epithelium. Paraffin sections of mammary gland tissue from control and *Mmtv-Ccl2* were stained with anti-macrophage-specific F4/80 antibody to detect macrophages in the stroma surrounding ductal (**a**, **b**) and alveolar epithelium (**c**, **d**) of mammary glands from adult control and *Mmtv-Ccl2* mice. Macrophages are indicated by *black arrows*. The number of F4/80-positive macrophages was quantified and represented as F4/80-positive macrophages/mm^2^ (**e**); *n* = 6 per group. Data are presented as mean + SEM with statistical analysis conducted using the unpaired *t* test, *p < 0.05

F4/80 immunohistochemistry was performed to investigate the effect of epithelial cell-derived CCL2 on the number of macrophages in the mammary gland. F4/80-positive macrophages were observed in close proximity to ductal and alveolar epithelium in the mammary glands from both control (Fig. 2a and c, respectively) and *Mmtv-Ccl2* mice (Fig. 2b and d, respectively). No positive staining was observed in the mammary glands stained with isotype-matched irrelevant antibody (not shown). The density of macrophages in regions surrounding both ductal and alveolar epithelium was increased in *Mmtv-Ccl2* mice compared with control (Fig. 2e).

### CCL2 overexpression perturbs mammary gland morphogenesis during the estrous cycle

**Fig. 3:**
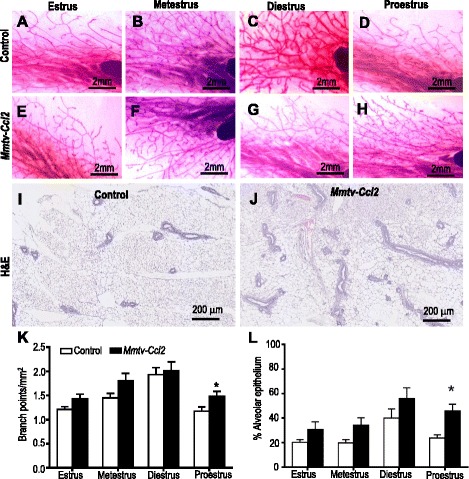
The effect of CCL2 overexpression on mammary gland morphogenesis during ovarian cycle. Mammary glands from control and *Mmtv-Ccl2* mice were whole-mounted and stained with carmine alum at estrus (**a**, **e**), metestrus (**b**, **f**), diestrus (**c**, **g**) and proestrus (**d**, **h**), respectively. The number of branch points per millimetre was calculated (**k**). Sections of paraffin-embedded mammary glands from control and *Mmtv-Ccl2* mice at the four stages of the cycle were H&E-stained (**i**, **j**, respectively, only proestrus stage shown) and alveolar epithelium quantified (**l**); *n* = 6–9 per group. Data are presented as mean + SEM with statistical analysis conducted using the unpaired *t* test, *p < 0.05

CCL2 overexpression did not affect estrous cyclicity. Estrous cycle length was (mean ± SEM) 5.4 ± 0.1 days for both *Mmtv-Ccl2* and control mice (n = 29 per genotype). There was no significant difference between the two groups in the percentage of time spent in each of the four stages of the estrous cycle. To assess the impact of CCL2 overexpression on mammary gland development across the estrous cycle, adult mammary glands from control and *Mmtv-Ccl2* transgenic mice were dissected at the different stages of the cycle and compared morphologically and histologically (Fig. 3). Despite normal secondary branching and alveolar architecture during the development phase of the cycle (metestrus and diestrus) in transgenic mice, *Mmtv-Ccl2* mice exhibited increased density of branch points (Fig. 3k) and an increase in the tissue area comprised by alveolar epithelium (Fig. 3l) at the proestrus phase of the cycle, suggesting a perturbation in the cyclic regression of the mammary gland. Thus, mammary gland function at the proestrus phase of the cycle was investigated in further experiments.

**Fig. 4:**
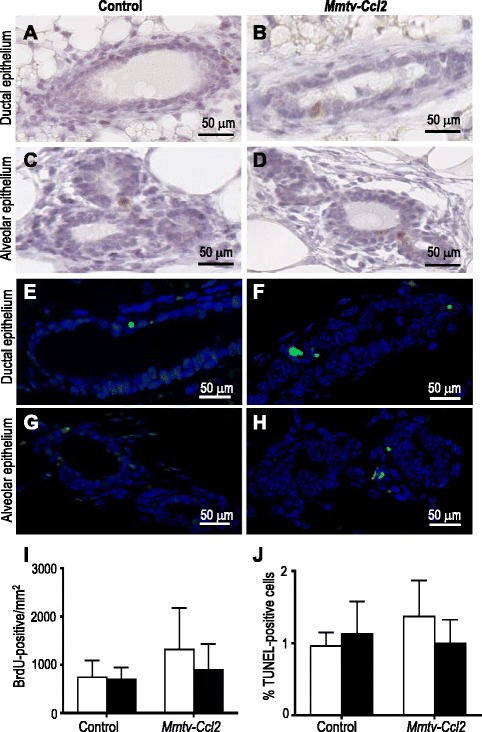
The effect of CCL2 overexpression on mammary epithelial cell proliferation and cell death. Paraffin-embedded sections of mammary gland tissue from control and *Mmtv-Ccl2* mice at proestrus were stained with anti-bromodeoxyuridine (BrdU) antibody to detect proliferating ductal (**a** and **b**) and alveolar (**c** and **d**) epithelial cells. The number of BrdU-positive cells (*brown*-stained cells) within ductal and alveolar epithelium was calculated and expressed as BrdU-positive cells/mm^2^ (**i**). Paraffin-embedded sections of mammary gland tissue from control and *Mmtv-Ccl2* mice at proestrus were stained with TUNEL to detect dying ductal (**e** and **f**) and alveolar (**g** and **h**) epithelial cells. The percent of TUNEL-positive cells (*green*-stained cells) within ductal and alveolar epithelium was calculated (**j**); *n* = 6 per group. Data are presented as mean + SEM with statistical analysis conducted using the unpaired *t* test, *p < 0.05

To investigate the effect of CCL2 on epithelial cell turnover at the proestrus phase of the cycle, mammary glands from *Mmtv-Ccl2* and control mice were stained for BrdU, indicative of cell proliferation, and TUNEL, indicative of cell death. BrdU-positive cells were observed in both ductal and alveolar epithelium of mammary glands from control (Fig. 4a and c, respectively) and *Mmtv-Ccl2* (Fig. 4b and d, respectively) mice. There was no significant difference in the density of proliferating ductal or alveolar epithelial cells in *Mmtv-Ccl2* compared to control mice (Fig. 4i). TUNEL-positive cells were observed in both ductal and alveolar epithelium of mammary glands from control (Fig. 4e and g, respectively) and *Mmtv-Ccl2* (Fig. 4f and h, respectively) mice. There was no significant difference in the density of TUNEL-positive ductal or alveolar epithelial cells in *Mmtv-Ccl2* compared to control mice (Fig. 4j).

**Fig. 5:**
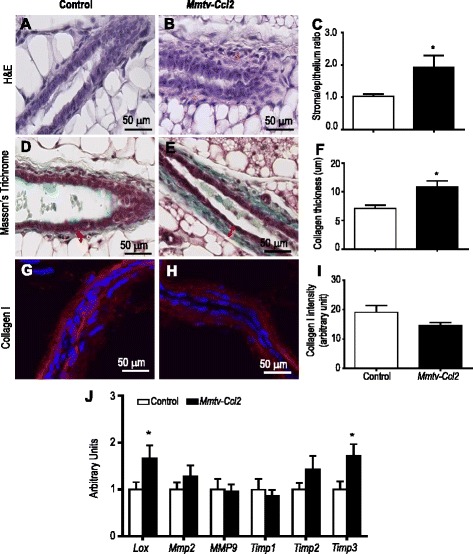
The effect of CCL2 overexpression on abundance of stroma and collagen. Sections of mammary gland tissue from control (**a**, **d**, **f**) and *Mmtv-Ccl2* (**b**, **e**, **h**) mice at proestrus were stained with H&E (**a**, **b**), Masson’s trichrome (**c**, **d**) and collagen I antibody (**g**, **h**) and quantified (**c**, **f**, **i**); *n* = 6 per group. Data are presented as mean + SEM with statistical analysis conducted using the unpaired *t* test, *p < 0.05 compared to control. Mammary glands from both groups of mice (*n* = 8) were dissected and frozen in liquid nitrogen. Messenger RNAs of collagen remodelling enzymes including *Lox*, *Mmp2*, *Mmp9*, *Timp1*, *Timp2* and *Timp3* were extracted and measured by RT-PCR. The amount of mRNA was normalised to *Actb* expression, and is given in arbitrary units, where the average of the control is 1 (**j**). Data are presented as mean + SEM with statistical analysis conducted using the unpaired *t* test; *p < 0.05 compared to control. *LOX* lysyl oxidase, *MMP* matrix metalloproteinase, *TIMP* tissue inhibitor of matrix metalloproteinases

Upon further examination of H&E-stained thin sections, an increased thickness of stroma surrounding epithelial ducts was evident in the mammary glands of transgenic mice. To investigate this, the ratio of stroma to epithelium was quantified in H&E-stained sections from control and *Mmtv-Ccl2* mice at proestrus (Fig. 5a and b, respectively). There was a twofold increase in the area of stroma relative to epithelium in *Mmtv-Ccl2* mice (Fig. 5c). The increase in stroma in CCL2-overexpressing mice was further investigated by analysing the extracellular matrix. The thickness of collagen, assessed by Masson’s trichrome stain, in control and *Mmtv-Ccl2* (Fig. 5d and e, respectively) mice was increased (Fig. 5f) however the abundance of monomeric collagen 1 detected by collagen I antibody staining was comparable in *Mmtv-Ccl2* mice (Fig. 5g-i). Quantitative RT-PCR was used to analyse the expression of mRNA encoding enzymes that are involved in collagen remodelling. The abundance of both lysyl oxidase (*Lox*) and tissue inhibitor of metalloproteinase 3 (*Timp3*) mRNA were increased in mammary glands of *Mmtv-Ccl2* mice compared to control mice, while matrix metalloproteinase 2 (*Mmp2*), *Mmp9*, *Timp1* and *Timp2* mRNA were not significantly altered (Fig. 5j). Combined, these results suggest that CCL2 overexpression causes increased abundance of stroma surrounding epithelium through perturbation of remodelling of the extracellular matrix.

### CCL2 overexpression increases susceptibility to DMBA-induced mammary gland cancer

**Fig. 6:**
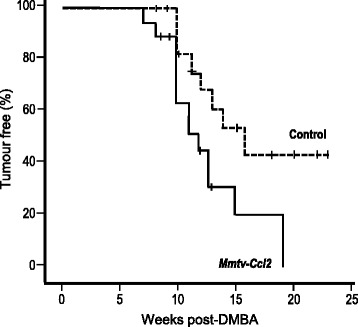
The effect of CCL2 overexpression on mammary gland cancer susceptibility in mice. Kaplan–Meier survival plot showing the percentage of tumour-free mice following 7,12-Dimethylbenz(a)anthracene (*DMBA*) treatment in weeks for control and *Mmtv-Ccl2* mice; n = 18 per group. In *Mmtv-Ccl2* mice, the incidence of mammary tumours was increased (*p* = 0.04 chi squared) and mammary tumour-free survival was reduced (*p* = 0.025 log rank) compared to control mice

To investigate the effect of CCL2 overexpression on mammary gland cancer susceptibility, control and *Mmtv-Ccl2* mice were challenged with the chemical carcinogen DMBA. Tumour free survival was analysed on a Kaplan–Meier plot (Fig. 6). The incidence of mammary tumours was higher in *Mmtv-Ccl2* mice, with 14 of 18 mice (78%) developing a mammary gland tumour compared to 8 of 18 (44%) control mice developing a mammary gland tumour (*p* = 0.04). There was also a significant decrease in mammary tumour-free survival in *Mmtv-Ccl2* mice compared to control mice (*p* = 0.025); mean latency (mean ± SD) was 12.7 ± 1.0 weeks in *Mmtv-Ccl2* mice, and 16.7 ± 1.4 weeks in control mice.

Analysis of a subset of the tumours revealed the majority represented adenosquamous carcinomas, with early mammary intraepithelial neoplasia and other carcinoma types present (Table 1). The mitotic index and inflammation score varied widely between tumours, and there was no significant difference in these parameters between control and *Mmtv-Ccl2* mice.

**Table 1:** Characteristics of DMBA-induced tumours from control and *Mmtv-Ccl2* mice

Genotype	Latency (weeks)	Histopathology	Mitotic index	Inflammation
*Control*	13	Spindeloid carcinoma	0	2
	10	Adenosqaumous carcinoma	3	5
	14	Adenosqaumous carcinoma	2	2
	16	Early MIN	0	0
	10	Adenosquamous carcinoma	N/A	4
	12	Adenosquamous carcinoma	1	4
	11	Carcinosarcoma	0	2
	10	Early MIN	0	1
*Mmtv-Ccl2*	10	Acinar	0	0
	11	Adenosquamous carcinoma	N/A	2
	10	Adenosquamous carcinoma	2	5
	19	Early MIN	0	0
	10	Adenosqaumous carcinoma	0	2
	13	Glandular carcinoma	0	2
	13	Cribiform carcinoma	1	4
	8	Adenosquamous carcinoma	1	5
	19	Early MIN	0	0

Latency in weeks after final 7,12-Dimethylbenz(a)anthracene (DMBA) administration. Mitotic index was determined by counting the number of mitotic cells in a × 10 field of view. Inflammation was scored as 0 (none), 1 (mild, focal), 2 (mild, multifocal), 3 (moderate, focal), 4 (moderate, multifocal) or 5 (marked, diffuse or multifocal). *MIN* mammary intraepithelial neoplasia, *N/A* not assessed

### Epithelial cell CCL2 is increased in breast tissue of high mammographic density in women

**Fig. 7:**
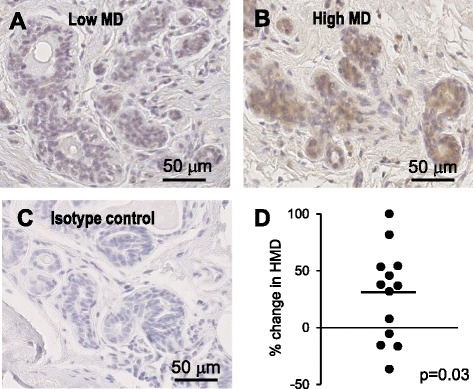
CCL2 in paired samples of high and low mammographic density (*MD*). CCL2 immunostaining of human breast tissue of low MD (**a**) and high MD (**b**) and isotype-matched negative control (**c**). Intensity of staining within the epithelium was quantified and data are presented as percent change in abundance of CCL2 in high density tissue compared to low density tissue for each paired sample; *horizontal bar* indicates the mean (**d**) analysis by paired sample *t* test (*n* = 13). *HMD* high mammographic density

The increase in stroma, collagen and mammary cancer risk in *Mmtv-Ccl2* mice resembled key clinical and histological features of HMD in women. To investigate whether epithelial cell-derived CCL2 might be associated with HMD, CCL2 was quantified in paired breast tissue samples biopsied from regions of high and low density from women undergoing prophylactic mastectomy. CCL2 staining was observed in the epithelium of human breast tissue of low and high mammographic density, while no staining was present in isotype-matched antibody control sections (Fig. 7). A few cells within the stromal compartment also stained positive for CCL2; however, the abundance of these scarce cells did not appear to be affected by density. Abundance of CCL2 in epithelial cells, as measured by intensity of CCL2 staining, was increased in tissue with HMD compared to the paired LMD tissue from the sample patient (*p* = 0.03).

## Discussion

This study investigated the significance of CCL2 in regulating macrophage recruitment, healthy mammary gland development, and the risk of cancer. Overexpression of CCL2 by the mammary gland epithelium was achieved through generation of a transgenic mouse model wherein *Ccl2* mRNA expression was under the control of the mammary epithelial cell-specific promoter MMTV. Consistent with the known role of CCL2 as a macrophage chemoattractant [36, 37], constitutive expression of epithelial cell-specific CCL2 resulted in increased abundance of stromal macrophages in the mammary gland. Therefore, this mouse model enabled the examination of the effect of CCL2 on macrophage infiltration, collagen remodelling, cyclic mammary gland regression and DMBA-induced cancer risk.

### CCL2 overexpression perturbs mammary gland regression and collagen remodelling

In cycling non-pregnant mice, the mammary epithelium undergoes ductal development and regression over the course of each ovarian cycle under the influence of ovarian hormones [31]. For the mammary gland to undergo these dynamic developmental processes, tight regulation of signals within the cytokine microenvironment that affect the balance between proliferation, differentiation, and apoptosis, and tissue breakdown and repair is required [8, 38]. Increased ductal branch points and abundance of alveolar buds observed in mice overexpressing CCL2 at the proestrus phase of the cycle suggests that despite normal estrous cyclicity, mammary gland regression was perturbed. However, at estrus, the mammary epithelium exhibited a normal basic ductal structure, implying that regression is delayed rather than completely inhibited.

Little is known of the role of CCL2 in mammary gland development and function; however, some studies suggest that it may be involved in mediating inflammation associated with the early response to tissue injury and post-weaning involution. Elevated expression of CCL2 is observed during the first stage of mammary gland involution, where it is believed to be involved in early recruitment of macrophages, and decreases during the later stage of involution when tissue remodelling peaks [39]. Lipopolysaccharide administered to the lactating mammary gland increases abundance of CCL2, which precedes macrophage recruitment and partial mammary gland involution [40]. These instances of acute elevation in CCL2 may form part of the immune signalling fingerprint that promotes an inflammatory response; however, later suppression of CCL2 may be required for appropriate tissue remodelling.

Mammary gland regression during the ovarian cycle involves a series of processes, including apoptosis of the mammary epithelial cells, clearance of dying epithelial cells by phagocytosis and remodelling of mammary extracellular matrix [7, 41]. Epithelial cell proliferation and apoptosis was unaffected by constitutive expression of the *Ccl2* transgene by mammary epithelium, which suggests that CCL2 might not affect epithelial cell turnover in the adult cycling mammary gland. However, the stromal area around ductal mammary epithelium was significantly larger in the presence of epithelial cell-derived CCL2, and therefore CCL2 might be involved in regulation of the process of stroma breakdown and repair necessary for cyclic remodelling.

Mammary gland stroma is composed of extracellular matrix proteins, and a wide variety of cells, including fibroblasts and leukocytes such as macrophages and eosinophils. Collagen is a prominent component of extracellular matrix in the mammary gland stroma that contributes significantly in supporting tissue integrity and elasticity; furthermore; the deposition and remodelling of collagen has been found to be highly associated with normal mammary gland development and tumourigenesis [42–44]. Increased *Timp3* mRNA expression was observed in CCL2 overexpressing mice, indicating CCL2 may enhance the inhibitory effect of TIMPs on MMP-mediated collagen degradation, resulting in elevated collagen deposition. Moreover, high expression of epithelial cell-derived CCL2 also causes increased expression of lysyl oxidase mRNA (*Lox*), a collagen crosslinking enzyme involved in crosslinking elastin and collagen in extracellular matrix and hence, enhancing tissue integrity [44]. This could also contribute to the increased collagen deposition around mammary epithelium in the presence of abundant CCL2, as increased LOX expression could lead to increase mammary gland stromal stiffness and collagen crosslinking, which could further inhibit MMP-mediated collagen degradation. Although mRNA for collagens and other collagen biosynthesis proteins were not assessed, their coordinated upregulation is feasible.

CCL2-driven inflammation is known to promote fibrosis and collagen deposition in a number of disease states, including human pulmonary fibrosis [45], bleomycin-induced pulmonary fibrosis in rats and mice [46, 47], and interstitial renal fibrosis in mice [48]. Macrophages are an essential mediator of inflammation-associated fibrosis, and have been shown to promote collagen synthesis in vitro [49–51]. In the mammary gland, macrophages promote collagen fibrillogenesis as a critical part of healthy pubertal development [2]. Therefore, increased abundance of mammary gland macrophages, stroma and collagen in CCL2 overexpressing mice is consistent with the known role of CCL2 in driving inflammation and collagen deposition.

These findings suggest that CCL2-driven inflammation increases collagen deposition and inhibits collagen remodelling around the mammary epithelium. Through promotion of macrophage recruitment and the activities of TIMP3 and LOX, high CCL2 expression would be expected to cause imbalance in the turnover of collagen, which in turn could impair mammary gland regression during the proestrus phase of the ovarian cycle.

### CCL2-driven breast cancer risk

It is well-established that chronic inflammation increases cancer risk through oncogenic mutations, genome instability, early tumour promotion, and enhanced angiogenesis [13, 14]. Chronic inflammation leads to collagen deposition and fibrosis associated with increased LOX expression [52] and increased stromal collagen deposition has also long been recognised as a major contributing factor to the increased mammographic density that increases risk of breast cancer [23, 25, 53, 54]. This study suggests that expression of epithelial cell-specific CCL2 by mammary epithelium increases mammary cancer susceptibility in mice through inducing a low level of chronic inflammation in the mammary gland, characterised by increased macrophage infiltration and fibrosis associated with perturbed collagen deposition and remodelling. However, further studies to dissect the contribution of macrophages to this phenotype are required to establish their role in the increased mammary cancer risk observed in *Mmtv-Ccl2* transgenic mice.

Population studies support the notion that increased CCL2 expression increases breast cancer risk and cancer progression. The risk of developing metastasis is significantly increased in patients with breast cancer who carry the CCL2-2518 A/G promoter polymorphism, compared to A/A homozygotes [55]. The A/G genotype causes increased CCL2 production in monocytes after stimulation compared to the A/A genotype [56] and may similarly affect CCL2 expression by mammary epithelium. However, it is unknown whether the protein concentration of CCL2 in the transgenic mice is similar or above the physiological concentration of CCL2 that might be present in the human breast. In addition, altered responsiveness to CCL2 through polymorphism in the receptor CCR2 also affects breast cancer susceptibility. The CCR2-64I polymorphism, which slightly impairs capacity to transduce CCL2 signals, is underrepresented in patients with breast cancer [17, 57], and this might provide protection from tumour formation through reduced recruitment of tumour-promoting macrophages.

High mammographic density is associated with fourfold to six-fold increased breast cancer risk in the highest versus lowest quartiles, and the association remains after correction for BMI and age [26]. Although the biological mechanisms that regulate mammographic density are poorly understood, HMD is associated with increased stroma, collagen deposition and TIMP3 protein compared to tissue of LMD [58–60]. These biological characteristics are strikingly similar to the mammary glands of *Mmtv-Ccl2* transgenic mice, in which overexpression of CCL2 caused increased abundance of stroma, collagen thickness, mRNA encoding TIMP3 and elevated risk of DMBA-induced mammary cancer. An inflammatory basis for HMD is suggested by studies in paired samples of high and low mammographic density tissue. Tissue with HMD exhibits elevated inflammatory COX2, increased number of vimentin-positive immune cells within the epithelium, and reduced abundance of CD206-positive “alternatively activated” macrophages compared to paired breast tissue samples of LMD [25, 61, 62], and the increase in CCL2 reported here. Based on these observations, we propose that CCL2-driven inflammation might play a role in increasing mammographic density and breast cancer risk.

The potential role of inflammation in driving high mammographic density suggests that use of non-steroidal anti-inflammatory drugs (NSAID), which are already known to reduce breast cancer risk [63, 64], could be effective in reducing mammographic density. In a mouse model of HMD, Celecoxib, a selective NSAID that specifically inhibits COX2, was shown to reduce collagen deposition in the mammary gland, and reduce tumour development and metastasis [65]. Although there appears to be no association between NSAID use and mammographic density [66], a study of nearly 30,000 postmenopasual women found that NSAID use reduced the risk of increasing mammographic density over a period of 9–28 months [67]. Further studies investigating the potential for NSAID to reduce breast cancer risk associated with HMD are required.

## Conclusion

Constitutive expression of CCL2 by the mouse mammary epithelium induces a state of low level chronic inflammation that perturbs collagen remodelling and elevates cancer risk. We propose that CCL2-driven inflammation contributes to the increased risk of breast cancer observed in women with HMD. Future studies will examine how CCL2 is regulated in the mammary gland, and the biological function of CCL2 in increasing mammographic density in women. Understanding the underlying cellular mechanisms of how inflammation affects breast function will shed light on cancer susceptibility and may uncover new opportunities to therapeutically reduce breast cancer risk.

## Abbreviations

Ampr: ampicillin-resistance gene
BMI: body mass index
bp: base pairs
BrdU: bromodeoxyuridine
CCL2: CC-chemokine ligand 2
CCR2: CCL2 receptor
DAB: 3,3 diaminobenzadine
DAPI: 4’,6-diamidino-2-phenylindol
DMBA: 7,12-Dimethylbenz(a)anthracene
ELISA: enzyme-linked immunosorbent assay
HMD: high mammographic density
LMD: low mammographic density
LOX: lysyl oxidase
MMP: matrix metalloproteinase
MMTV: mouse mammary tumour virus 206 promoter
NSAID: non-steroidal anti-inflammatory drugs
PFA: paraformaldehyde
SEM: standard error of the mean
TIMP: tissue inhibitor of matrix metalloproteinases
TUNEL: terminal deoxynucleotidyl transferase dUTP nick end labelling
